# Association Between Alterations in Penicillin-Binding Proteins and β-Lactam Resistance in *Ligilactobacillus salivarius*

**DOI:** 10.3390/ijms27219683

**Published:** 2026-10-29

**Authors:** Marta Dec, Tomasz Hauschild, Renata Urban-Chmiel

**Affiliations:** 1 Department of Veterinary Prevention and Avian Diseases, Faculty of Veterinary Medicine, University of Life Sciences in Lublin, Głęboka 30 Street, 20-612 Lublin, Poland; renata.urban@up.edu.pl; 2 Department of Microbiology and Biotechnology, Faculty of Biology, University of Bialystok, Ciołkowskiego Street 1J, 15-245 Bialystok, Poland; thausch@uwb.edu.pl

**Keywords:** *Ligilactobacillus salivarius*, beta-lactam resistance, penicillin-binding proteins

## Abstract

The aim of this study was to elucidate the molecular basis of β-lactam resistance in *Ligilactobacillus salivarius*, with a focus on mutations in genes encoding penicillin-binding proteins (PBPs). Twelve *L. salivarius* strains (2 reference strains and 10 turkey-derived isolates) were analyzed. Susceptibility to β-lactams was determined by the broth microdilution method. Whole-genome sequencing (WGS) analyses and polymerase chain reaction (PCR) were used to detect resistance genes. Alterations in 3 PBPs (FtsI, PBP2x-like, and PBP1A) were identified by comparative analysis of presumptive amino acid (aa) sequences. Phylogenetic relationships between strains were assessed using multilocus sequence typing (MLST). Six strains were resistant to ampicillin (MIC ranging from 32 to ≥128 mg/L) and showed elevated MICs for cephalosporins. No β-lactamase genes were detected in any strain. Resistant strains exhibited distinct patterns of aa alterations in the transpeptidase domains of FtsI and PBP2x-like proteins, whereas susceptible strains with elevated ampicillin MICs (2–4 mg/L) showed only partial alteration profiles. PBP1A displayed limited variation and no clear association with resistance phenotype. Taken together, these findings are consistent with an association between β-lactam resistance in *L. salivarius* and alterations in high-molecular-weight (HMW) PBPs, particularly FtsI and PBP2x-like proteins, in the absence of detectable β-lactamase production. However, further studies, including functional analyses, are needed to clarify the role of these PBP alterations in the observed resistance phenotype.

## 1. Introduction

*Ligilactobacillus salivarius* is a Gram-positive lactic acid bacterium naturally colonizing the gastrointestinal tract of humans and animals. Its presence has also been documented in the oral cavity, vagina, and human breast milk, as well as in selected food products [1]. *L. salivarius* strains exhibit strong epithelial adhesion and antimicrobial and antioxidant activity and frequently produce bacteriocins with pronounced antagonistic properties. In vivo and ex vivo studies have demonstrated multiple beneficial effects of *L. salivarius*, including gut microbiota modulation, immune stimulation, and improved poultry performance [2,3,4,5]. Owing to these properties, selected *L. salivarius* strains are applied as probiotics in both human medicine and veterinary practice [3]. According to current regulatory guidelines (EFSA, 2025) [6], probiotic strains must meet strict safety criteria, including defined antimicrobial susceptibility profiles and the absence of acquired resistance genes. At the same time, intrinsic or non-transferable resistance may be advantageous, as it enables probiotic strains to survive during antibiotic therapy and maintain their functional activity within the host.

Although members of the family *Lactobacillaceae*, including *Ligilactobacillus salivarius*, are generally regarded as beneficial commensals, they have occasionally been implicated in opportunistic infections, particularly in immunocompromised individuals and patients with severe underlying diseases. In a retrospective study conducted at a university-affiliated hospital in northern Taiwan, which included 89 cases of *Lactobacillaceae* bacteremia diagnosed between 2000 and 2014, *L. salivarius* was the most frequently isolated species. The predominant sources of infection were intra-abdominal infections and catheter-related bloodstream infections [7]. Traditionally, penicillins have been considered the treatment of choice for bacteremia and infective endocarditis caused by *Lactobacillaceae*, sometimes in combination with an aminoglycoside [8,9]. Therefore, a better understanding of the mechanisms underlying β-lactam resistance in *L. salivarius* is important not only for probiotic safety assessment but also for the management of the rare cases of invasive infections caused by this species.

β-lactam antibiotics exert their antibacterial activity by inhibiting cell wall biosynthesis through binding to enzymes called penicillin-binding proteins (PBPs) that are responsible for the polymerization of peptidoglycan precursors and the cross-linking of glycan strands [10]. Members of the *Lactobacillaceae*, including *L. salivarius*, are generally considered susceptible to β-lactam antibiotics, with resistance being reported only sporadically [1,11,12,13,14]. The molecular mechanisms underlying β-lactam resistance in this bacterial family remain poorly understood. Rare reports have described the presence of genes encoding extended-spectrum β-lactamases (ESBLs) in *Lactobacillaceae* (other than *L. salivarius*) [15,16,17,18], as well as alterations in PBPs [19]. Resistance may also develop as a result of prolonged exposure of *Lactobacillaceae* to β-lactam antibiotics, which can promote adaptive responses such as changes in protein homeostasis, stress response pathways, and cellular metabolism [20]. Notably, in *L. salivarius*, the molecular basis of β-lactam resistance has not yet been elucidated, but it has been shown not to be associated with β-lactamase production [1,13]. In other Gram-positive bacteria such as *Staphylococcus* spp. and *Streptococcus* spp., resistance to β-lactam antibiotics is most commonly mediated either by antibiotic degradation via β-lactamases or by structural alterations in PBPs that reduce antibiotic affinity while preserving essential enzymatic activity. In some species, resistance may also involve the acquisition of genes coding for low-affinity PBPs (such as PBP2a in MRSA) or cell wall thickening that impairs antibiotic penetration [10].

In PBP-mediated resistance, HMW PBPs are primarily involved, including class A bifunctional enzymes possessing both transglycosylase and transpeptidase activities, as well as class B monofunctional transpeptidases. Importantly, resistance-associated substitutions leading to reduced β-lactam affinity are typically located within the transpeptidase domain [10,21].

This study builds on our earlier research, in which we demonstrated the occurrence of ampicillin-resistant *L. salivarius* strains in turkeys [1]. The present study extends this work by investigating the association between β-lactam susceptibility profiles and amino acid alterations in three putative PBPs. To the best of our knowledge, this aspect has not previously been investigated in *L. salivarius*, and the findings provide new insights into the potential molecular mechanisms underlying β-lactam resistance in this species.

## 2. Results

### 2.1. Antimicrobial Susceptibility of L. salivarius Strains and Detection of Resistance Genes

Among the 12 examined *L. salivarius* strains, 6 were classified as susceptible to AMP. Four of these strains, including the 2 reference strains, exhibited very low AMP MICs (≤0.5 mg/L), whereas 2 field isolates showed slightly higher MIC values ranging from 2 to 4 mg/L. The remaining 6 isolates were resistant to AMP, with MICs ranging from 32 mg/L to ≥128 mg/L. AMP-resistant isolates also demonstrated high MICs for amoxicillin/clavulanic acid (32 to ≥128 mg/L) and elevated MICs for first-, second-, third-, and fourth-generation cephalosporins compared with MICs for AMP-susceptible strains. Particularly high MIC values for cefuroxime (2G) (≥128 mg/L) and ceftiofur (3G) (64 mg/L) were observed for strain T25a. No differences in the observed imipenem MIC values (MIC ≤ 2 mg/L) were detected among the tested strains within the concentration range examined (Table 1).

None of the 12 *L. salivarius* strains carried any of the resistance genes tested by PCR, i.e., *blaZ, blaTEM, blaOXA, blaCTX,* and *blaSHV*. Likewise, WGS analysis did not reveal the presence of known acquired β-lactam resistance determinants in any of the studied strains (*n* = 6), although genes conferring resistance to other classes of antibiotics were detected (Table 2).

### 2.2. Classification of Putative PBPs

Based on genome annotations, three genes encoding putative PBPs—FtsI (688 aa), PBP2x-like (718 aa), and PBP1A (714 aa)—were identified in the analyzed *L. salivarius* strains (T2a, T17f, T21a, T25a, LMG 9476, and LMG 9477). The calculated mol. wt of these proteins are 75, 78, and 79 kDa, respectively, which indicates that they belong to HMW PBPs (LMW PBPs called class C PBPs usually have molecular weights below 50 kDa [22]). Conserved motifs characteristic of the transpeptidase domain were detected in the C-terminal regions of all three proteins, while additional sequence motifs typical of the glycosyltransferase domain were identified in the N-terminal region of PBP1A (Tables S1–S3). This suggests that FtsI and PBP2x-like proteins represent class B PBPs, whereas PBP1A is a class A PBP. BLAST analysis revealed 35% sequence similarity between FtsI and PBP2b (class B) of *Streptococcus pneumoniae* and between the 718-aa protein and PBP2x of *S. pneumoniae*. Domain annotation of the 718-aa protein using the NCBI Conserved Domain Database identified a region assigned to the strep_PBP2X family, as well as two C-terminal PASTA domains, further supporting its classification as a PBP2X-family protein. Accordingly, this protein was designated as PBP2x-like in the present study. In comparison, *L. salivarius* PBP1A exhibited 45% sequence similarity to PBP1A of *S. pneumoniae*, a representative class A PBP.

### 2.3. Mutations in L. salivarius PBPs and Their Association with β-Lactam Susceptibility

Comparative analysis of putative aa sequences of three PBP family proteins revealed clear differences between PBPs of AMP-susceptible and AMP-resistant *L. salivarius* strains, indicating a strong association between mutations in PBP-encoding genes and the observed β-lactam resistance phenotype.

Analysis of the FtsI transpeptidase sequences (688 aa) revealed 15 variable aa positions among the examined *L. salivarius* strains. All AMP-resistant strains shared a distinct mutation pattern comprising multiple substitutions located within the C-terminal region of the protein. These included substitutions located between 525 and 663 aa, i.e., S525A, T620H, I631N, S660N, S661G, V662I, and N663D. Four of the six AMP-resistant strains carried an additional substitution Pro → Leu at position 405, while three strains exhibited an Ala → Val substitution at position 673. Furthermore, all phenotypically resistant strains exhibited deletion of lysine at position 659 (Table 3).

Certain aa substitutions were also observed among AMP-susceptible strains. In particular, strains T7c and T27, which displayed elevated AMP MIC values (2–4 mg/L), carried mutations at four positions, i.e., 525, 620, 631, and 662, that overlapped with mutation sites identified in AMP-resistant strains. However, at positions 620 (Thr → Asn) and 631 (Ile → Thr), the aa substitutions observed in T7c and T27 differed from those detected in resistant isolates (Table 3). No mutations were observed in the conserved motifs (SVVK, SSN, SIFK, STAK, and KTG) located between aa 387 and 618 (Table S1).

**Figure 1:**
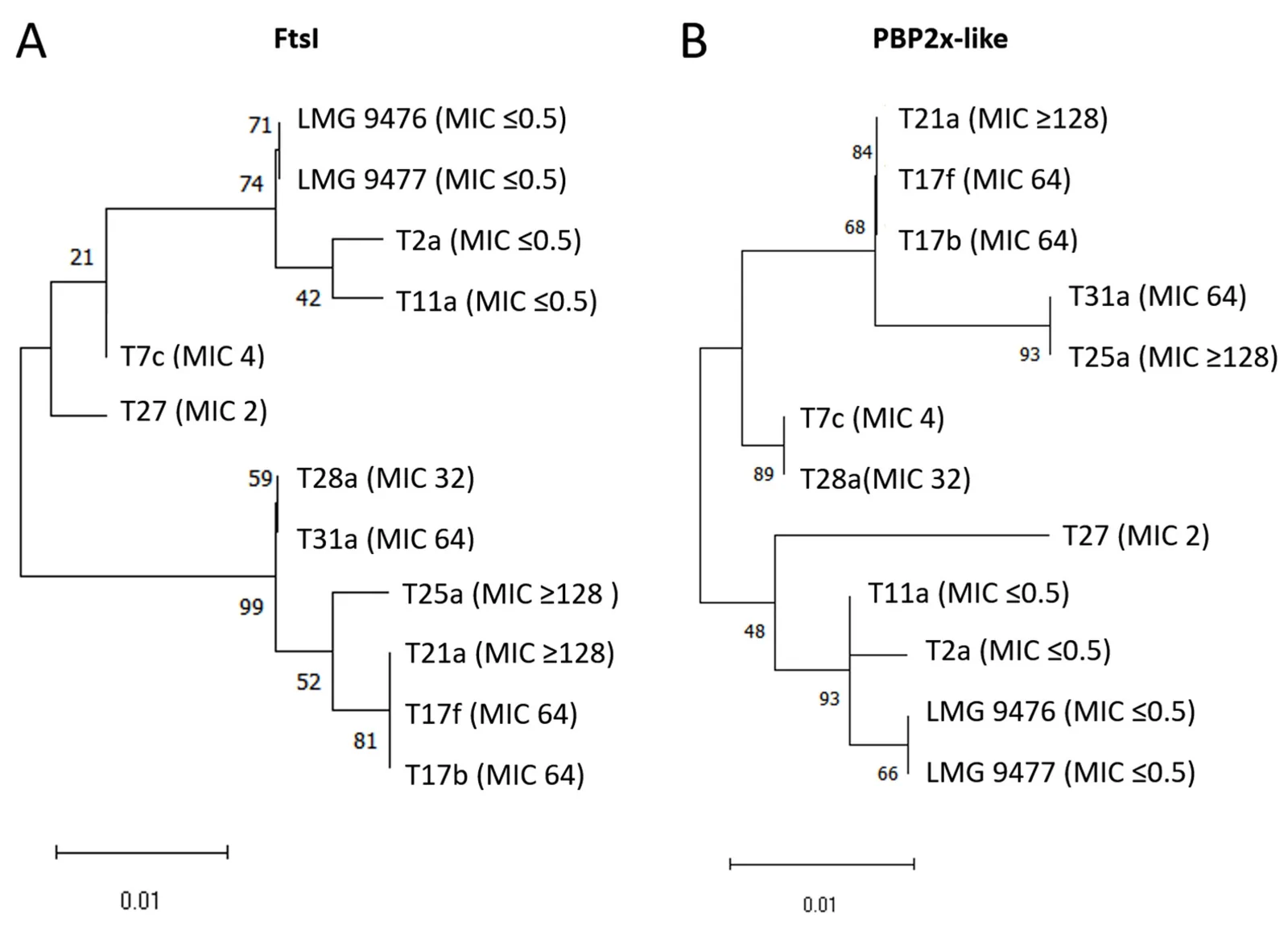
Dendrogram showing the similarity of the aa sequences of the FtsI protein (**A**) and the PBP2x-like protein (**B**) between the tested *L. salivarius* strains. The MIC of AMP is given in parentheses. The percentage of replicate trees in which the associated taxa were clustered together in the bootstrap test (300 replicates) is shown next to the branches. Scale bars show genetic distance.

The dendrogram illustrating the similarity of FtsI aa sequences showed two major clusters clearly separating phenotypically resistant and susceptible strains. Within the susceptible cluster, a distinct subcluster comprising strains T7c and T27 was observed, reflecting their reduced susceptibility to AMP (Figure 1A).

Analysis of the PBP2x-like protein revealed 14 variable amino acid positions located within the C-terminal region of the protein (aa 351–633). Five substitutions, i.e., S351P, Y360H, G437A, L510I, and A577D, were present in nearly all AMP-resistant strains and, in some cases, also in susceptible strains with elevated ampicillin MICs, i.e., T7c and T27 (Table 4). In both of these strains, an additional substitution at position 547 (Phe→Leu) was detected, whereas strain T27 harbored four further amino acid substitutions. Similarly to the FtsI protein, no mutations were identified within the conserved motifs in the PBP2x-like protein (Table S2).

Dendrogram analysis based on PBP2x-like protein aa sequences revealed the presence of two major clusters generally corresponding to phenotypically susceptible and resistant strains. However, the separation was not absolute. Notably, the AMP-resistant strain T28a grouped together with the susceptible strain T7c, which exhibited an elevated AMP MIC value, both sharing an identical PBP2x-like aa sequence (Figure 1B). At the same time, it should be noted that strain T28a exhibited a one-order-of-magnitude lower MIC value for AMP (32 mg/L) and a markedly lower MIC value for ceftiofur (4 mg/L) compared with the remaining resistant strains. Thus, although the PBP2x-like protein aa sequence was identical in strain T7c (susceptible with an elevated AMP MIC) and T28a (resistant), the two strains differed markedly in their MIC values for β-lactam antibiotics. This suggests that alterations in the FtsI protein, observed in strain T28a, may contribute substantially to the β-lactam-resistant phenotype.

In the PBP1A protein sequence (714 aa) of the analyzed *L. salivarius* strains (*n* = 6, based on WGS analysis), seven variable aa positions were identified. However, most of them were located within the N-terminal portion of the protein (aa 105–220), and they were not clearly associated with the degree of susceptibility of the strains to β-lactam antibiotics. An exception was the V367I substitution (C-terminal region) observed in strain T25a, which exhibited particularly high MICs for cefuroxime (2G) and ceftiofur (3G) (Table 5 and Table S3).

As the PBP1A sequence variation showed no apparent association with the β-lactam susceptibility phenotype among the strains analyzed by WGS, this protein was not investigated further in the remaining isolates. Therefore, the limited number of strains examined precludes firm conclusions regarding the contribution of PBP1A to β-lactam susceptibility in *L. salivarius*.

### 2.4. Phylogenetic Analysis Based on MLST

**Figure 2:**
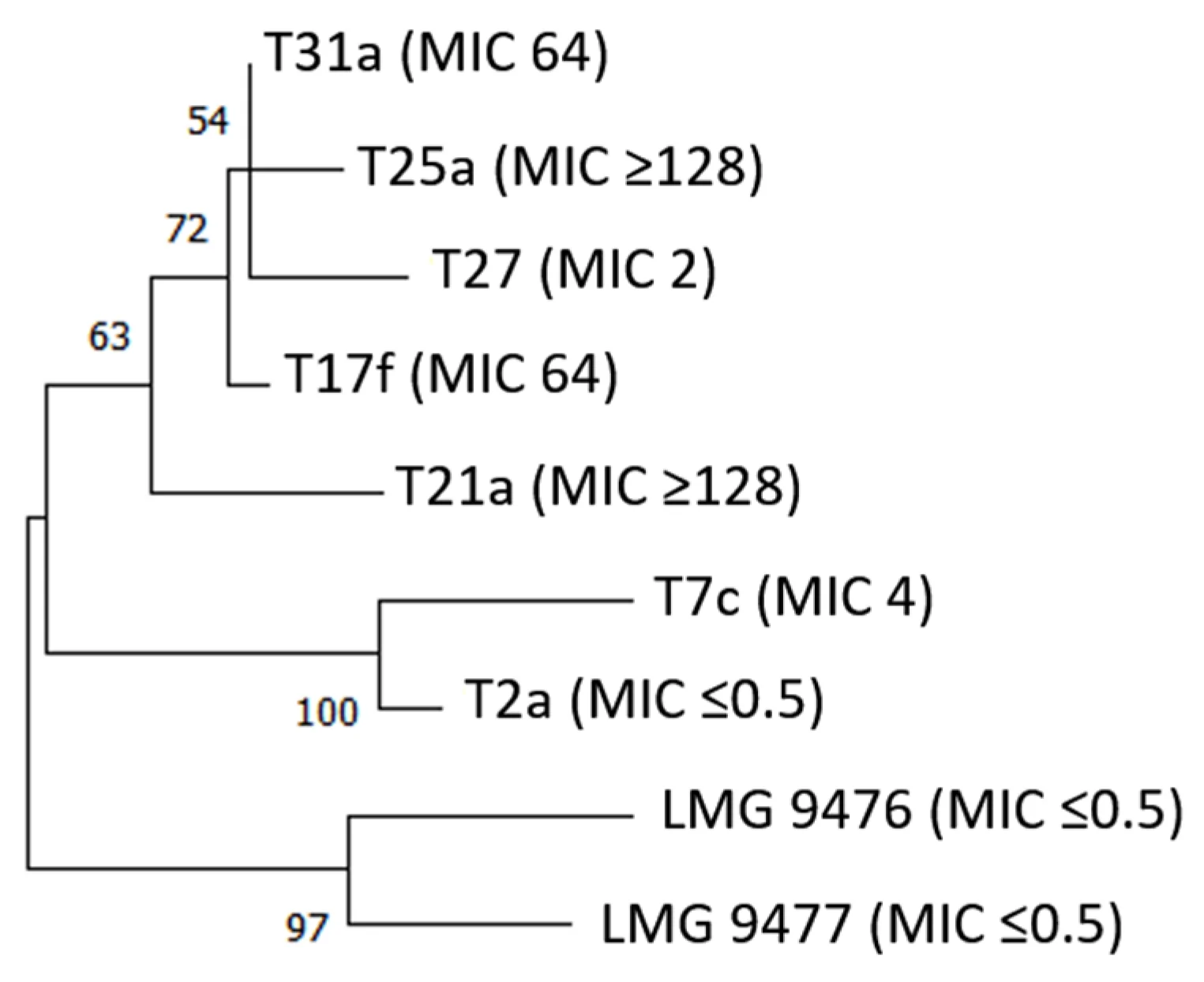
Phylogenetic relationships among *L. salivarius* strains inferred from concatenated sequences of five housekeeping genes (*pstB, rpoA, parB, nrdB*, and *rpsB*) using the Maximum Likelihood method implemented in MEGA ver. 11. The percentage of replicate trees in which the associated taxa were clustered together in the bootstrap test (300 replicates) is shown next to the branches. Scale bars show genetic distance. The MIC of AMP is given in parentheses. Strains T17b, T28a, and T11a were excluded from the MLST analysis because high-quality sequences were not obtained for all housekeeping genes.

Phylogenetic relationships among nine *L. salivarius* strains were inferred from concatenated sequences of five housekeeping genes (*pstB, rpoA, parB, nrdB*, and *rpsB*) (strains T17b, T28a, and T11a were excluded from the MLST analysis because high-quality sequences were not obtained for all housekeeping genes). The resulting dendrogram demonstrated genetic diversity of the strains and no evidence of a dominant clonal lineage (Figure 2). The two reference strains (LMG 9476 and LMG 9477), both susceptible to β-lactams, formed a well-supported cluster. Field isolates were distributed across multiple branches, with AMP-resistant strains (T31a, T25a, T17f, and T21a) and the susceptible isolate T27 with an elevated ampicillin MIC value forming a separate group. A similar clustering pattern was observed for the AMP-susceptible strain T2a and the susceptible isolate T7c with an elevated ampicillin MIC (Figure 2). Although the analysis revealed genetic diversity among the strains, the observed clustering of both resistant and susceptible isolates does not exclude shared ancestry or a contribution of clonal dissemination to the distribution of resistance-associated PBP variants.

## 3. Discussion

This study provides the first evidence that β-lactam resistance in *L. salivarius* is associated with alterations in HMW PBPs. Previously, using the nitrocefin assay, we demonstrated that resistance to AMP, accompanied by elevated MIC values for cephalosporins, was not associated with β-lactamase production in poultry-derived strains of *L. salivarius*, *Limosilactobacillus oris, Lactobacillus crispatus*, and *Lactobacillus johnsonii* [1]. Similar observations were reported by Campedelli et al. [13], who detected increased AMP MIC values (8–16 mg/L) in 18.5% (5/27) of strains belonging to the *L. salivarius* phylogroup but did not identify genes encoding β-lactamases in their genomes. It should be noted, however, that sporadic reports have described the presence of penicillinase [15] or extended-spectrum β-lactamase (ESBL) genes (*blaTEM, blaOXA-1, blaSHV, blaCTX-M-1,* and *blaCTX-M-2*) in some members of the *Lactobacillaceae* (other than *L. salivarius* or in strains not identified at the species level) [16,17,18]. Importantly, strains carrying these genes remained susceptible to AMP and exhibited variable susceptibility to cephalosporins [15,16].

To date, only one study has suggested that alterations in PBPs may underlie β-lactam resistance in *Lactobacillaceae*. Rosander et al. [19] identified point mutations in PBP1a, PBP2a, and PBP2x in the AMP-resistant strain *Lactobacillus reuteri* ATCC 55730. However, the AMP MIC was not reported for this strain or for the susceptible strain used in the comparative analysis [19].

The simultaneous occurrence of mutations in at least two HMW PBPs, namely FtsI and the PBP2x-like protein, observed in the β-lactam-resistant *L. salivarius* strains is consistent with reports from streptococci, where high-level resistance is often associated with alterations in more than one PBP [21,23]. Notably, the PBPs that undergo early modifications in *S. pneumoniae*, namely PBP2B and PBP2X, similarly to FtsI and PBP2X-like PBPs identified in this study, belong to class B HMW PBPs.

The findings of this study are consistent with the possibility of a stepwise development of β-lactam resistance in *L. salivarius*, potentially involving the accumulation of mutations in genes encoding PBPs and a gradual reduction in antibiotic susceptibility. Strains T7c and T27, which exhibited elevated AMP MICs and partial PBP alteration profiles, may represent intermediate phenotypes. However, this interpretation warrants further investigation using a larger number of strains.

The results also suggest that alterations in FtsI may play a prominent role in β-lactam resistance in *L. salivarius*, although this possibility remains to be confirmed through functional studies. In contrast, the contribution of PBP1A remains less clear. Among the six strains for which WGS data were available, including both AMP-susceptible and AMP-resistant isolates, no substantial alterations were identified in the C-terminal (transpeptidase) region of PBP1A, with the exception of a single substitution (V367I) detected in strain T25a. Although this strain exhibited particularly high MIC values for cefuroxime (2G) and ceftiofur (3G), it remains unclear whether V367I contributes directly to resistance, given its location away from the conserved catalytic motifs (Table S3). Nevertheless, studies in *S. pneumoniae* have shown that high-level resistance to cephalosporins (e.g., cefotaxime) requires alterations in PBP1A in addition to modifications in PBP2X [24]. These findings may be in line with previous reports demonstrating that not all PBPs contribute equally to β-lactam resistance and that specific PBPs may have a dominant role depending on the bacterial species [23]. For example, in *S. pneumoniae*, resistance to β-lactam antibiotics is primarily associated with alterations in PBP2B, PBP2X, PBP1A, and in some cases PBP2A, whereas the affinity of PBP1B and PBP3 for β-lactams remains largely unchanged [10].

The aa alterations identified in this study were located mainly in the C-terminal region of the PBP proteins, which contains the transpeptidase domain responsible for catalytic activity and β-lactam binding. Aa substitutions within this domain may alter the geometry of the active-site pocket, thereby reducing the affinity of PBPs for β-lactam antibiotics [10,21,23,25]. Interestingly, none of the β-lactam-resistant *L. salivarius* strains analyzed in this study carried mutations directly within the conserved catalytic motifs SXXK, SXN, and KTG that are essential for PBP enzymatic activity, and only a single substitution was located in the immediate vicinity of a catalytic motif, namely KTGT→KTGH in the FtsI protein. In many bacteria, resistance-associated substitutions in HMW PBPs are likewise located outside the catalytic motifs [10,21]. However, in some species, mutations have also been reported within these motifs. In *S. pneumoniae*, markedly elevated penicillin MIC values (from ≤0.03 up to 4 mg/L) have been associated with substitutions located both near and directly within the SXXK, SXN, and KTG/KSG motifs of PBP2B, PBP2X, and PBP1A [26]. Similarly, in *Enterococcus* spp., the highest β-lactam MIC values have been linked to substitutions affecting the catalytic serine residue within the SXXV motif of PBP5 [27].

The concurrent resistance to penicillins and cephalosporins observed in *L. salivarius*, while maintaining substantial susceptibility to carbapenems, is consistent with reports concerning other Gram-positive bacteria. Ubukata et al. [28] and Ohsaki et al. [29] demonstrated that simultaneous alterations in PBP1A, PBP2X, and PBP2B in *S. pneumoniae* were associated with a marked increase in MICs for penicillins and cephalosporins (e.g., AMP and cefotaxime from 0.08 up to 4 mg/L), accompanied by only a relatively moderate increase in MICs for carbapenems (imipenem from 0.004 up to 0.5 mg/L and panipenem from 0.03 up to 0.12 mg/L). A similar pattern appears to occur in *L. salivarius*. In our study, however, only two concentrations of imipenem (2 and 4 mg/L) were tested; therefore, potential differences in MIC values at lower carbapenem concentrations between the strains may not have been detected. Previous studies have reported imipenem MICs ranging from ≤0.03 to 0.5 mg/L for three *L. salivarius* isolates recovered from patients with bacteremia [30].

In intensive poultry farming, the frequent use of antimicrobial agents, particularly penicillins, may expose the gastrointestinal microbiota of turkeys to repeated β-lactam selection pressure. This could favor the emergence and selection of spontaneous mutations in genes encoding PBPs. Experimental evidence supporting this possibility was provided by Park and Rafi [31], who demonstrated that cultivation of *Clostridium perfringens* ATCC 13124 in media containing gradually increasing concentrations of penicillin, cephalothin, or ceftriaxone resulted in the selection of resistant mutants with altered PBPs.

The findings of the present study may be relevant both to the assessment of β-lactam susceptibility in clinical *L. salivarius* isolates and to the safety evaluation of strains intended for probiotic use. Given that this species has occasionally been associated with bacteremia [7,30], understanding the potential relationship between PBP sequence variation and β-lactam susceptibility may help interpret antimicrobial susceptibility profiles in clinical isolates. In the safety assessment of probiotic strains, whole-genome sequencing enables the screening of acquired antimicrobial resistance genes and may also allow the identification of aa alterations in PBPs that could be associated with reduced β-lactam susceptibility. The potential persistence of β-lactam-resistant probiotic strains in the gastrointestinal tract during antibiotic therapy should be considered alongside the risk that such strains could cause infections that may be difficult to treat, particularly in individuals with compromised immune function.

### Limitations of the Study

Although the findings of the present study are consistent with a potential PBP-associated mechanism of β-lactam resistance in *L. salivarius*, the contribution of individual amino acid alterations to the observed resistance phenotype remains to be confirmed through functional studies. In particular, direct assessment of β-lactam binding to mutated PBP variants—for example, using fluorescent penicillin analogues or biochemical binding assays—would provide valuable insight into the structural basis of reduced antibiotic affinity. Furthermore, analysis of PBP expression levels and potential compensatory changes in cell wall metabolism may help to elucidate the full spectrum of mechanisms contributing to β-lactam resistance in *L. salivarius*. Therefore, the observed associations between PBP mutations and resistance phenotypes should not be interpreted as direct proof of causality until validated by functional analyses.

An additional limitation of this study is that PBP1A sequence analysis was restricted to six strains for which WGS data were available. Although these included both ampicillin-susceptible and ampicillin-resistant isolates, the dataset was substantially smaller than that analyzed for FtsI and PBP2x-like proteins. Furthermore, the PBP1A sequences were not independently verified by Sanger sequencing. Therefore, conclusions regarding the contribution of PBP1A to β-lactam resistance should be interpreted with caution.

## 4. Materials and Methods

### 4.1. Bacterial Strains

Twelve *L. salivarius* strains were included in the study, comprising 2 reference strains, LMG 9476 (DSM 20554) and LMG 9477 (DSM 20555), and 10 wild-type isolates obtained from fecal samples of domestic turkeys originating from five commercial farms in Poland and collected between 2011 and 2014. The isolation procedure, species identification, and phenotypic and genotypic characterization of these isolates have been described previously [1,32], and the main criterion for the selection of these strains was their diverse susceptibility to ampicillin [1].

### 4.2. Antimicrobial Susceptibility Test

The susceptibility of *L. salivarius* strains to β-lactam antibiotics was determined using the broth microdilution method (two independent replicates). Testing with ampicillin (AMP) (0.5–128 µg/mL), cephalothin (0.125–64 µg/mL), cefuroxime (0.125–64 µg/mL), and ceftiofur (0.125–64 µg/mL) was performed manually according to a previously described protocol [1,6], whereas susceptibility to amoxicillin/clavulanic acid (0.5/0.25–32/16 µg/mL), imipenem (2–4 µg/mL), cefotaxime (single concentration 2 µg/mL), and cefepime (4–8 µg/mL) was determined using custom-made Sensititre ready-to-use microplates (Thermo Fisher, East Grinstead, UK). In both approaches, the same culture medium (ISO Sensitest supplemented with 10% MRS, Thermo Fisher Scientific, Basingstoke, UK), inoculum density, and incubation conditions (48 h) were applied. Classification of strains as AMP-resistant or AMP-susceptible was based on the EFSA guidance [6]. The remaining antimicrobial agents were not included in susceptibility categorization due to the lack of available interpretive criteria.

### 4.3. Whole-Genome Sequencing (WGS)

WGS of the reference strains LMG 9476 and LMG 9477, both susceptible to AMP, were retrieved from GenBank. Four field isolates, namely T17f, T21a, and T25a (AMP-resistant) and T2a (AMP-susceptible), were subjected to whole-genome nanopore sequencing using a PromethION device (Oxford Nanopore Technologies, Oxford, UK). Reads were down-sampled to 100× coverage using Rasusa v2.1.0, assembled using Flye v2.9.6 [33] with manual curation, polished using Medaka v2.1.0, and annotated using the NCBI Prokaryotic Genome Annotation Pipeline (PGAP) [34]. Whole-genome sequences have been deposited in the GenBank database under the following assembly accession numbers: GCA_054331695.1 (T2a), GCA_054331755.1 (T17f), GCA_054331715.1 (T21a), GCA_048572735.1 (T25a).

### 4.4. MLST

The MLST of *L. salivarius* strains was performed based on sequence analysis of five housekeeping genes, i.e., *pstB, nrdB, rpsB, rpoA*, and *parB*, following the approach described by Raftis et al. [35]. For strains not subjected to WGS, the genes were amplified by PCR [34], and amplicons were sequenced using the Sanger method (GenBank Acc. Nos. and allele Nos. listed in Table S4). Comparative analyses of the obtained DNA sequences were performed using the Maximum Likelihood method implemented in MEGA ver. 11.26 [36].

### 4.5. Detection of Resistance Genes

All isolates were screened for the resistance genes *blaZ, blaTEM, blaCTX, blaOXA,* and *blaSHV* by PCR using primers listed in Table S5. In the case of strains subjected to genomic sequencing, resistance genes were detected based on WGS analysis using ResFinder 4.1 [37] and Resistance Gene Identifier (RGI) ver. 6.0.3 [38].

### 4.6. Analysis of Mutations in Genes Encoding PBPs

Mutations were analyzed in three genes encoding putative PBPs, i.e., peptidoglycan D,D-transpeptidase FtsI family protein, penicillin-binding transpeptidase domain-containing protein (PBP2x-like), and PBP1A family penicillin-binding protein. The genes encoding FtsI and PBP2x-like protein were amplified from all 12 strains using the primers listed in Table 6. They were designed using Primer-BLAST (NCBI, https://www.ncbi.nlm.nih.gov/tools/primer-blast/, accessed on 10 August 2026), so that the amplified fragments correspond to the C-terminal region of the proteins, which includes the transpeptidase domain. In contrast, PBP1A sequence analysis was based exclusively on WGS data available for six strains.

Amplicons were sequenced using the Sanger method, and DNA sequences of PBP genes were deposited in GenBank (Acc. Nos. PZ015971-PZ015982). The aa sequences were predicted using the NCBI translate tool ORF finder (https://www.ncbi.nlm.nih.gov/orffinder/, accessed on 10 August 2026), and sequence alignment was performed using MEGA ver. 11 [35].

### 4.7. Detection and Classification of PBPs

Genes encoding PBPs in the *L. salivarius* strains tested were identified based on WGS data using annotated genome assemblies. The putative PBPs were classified as HMW or low-molecular-weight (LMW) and assigned to class A or class B based on protein length and domain organization. Identification of the transpeptidase domain was based on the presence of conserved motifs SXXK, SXN, and KTG/KSG, whereas the presence of an N-terminal glycosyltransferase domain characteristic of class A PBPs was inferred from conserved sequence signatures typical of GT51 enzymes (EDxxFxxHxG, STxxxQ, RKxxE, KxxIxxxYxN, and RxxxVL) [25,39].

## 5. Conclusions

This study provides the first evidence of an association between mutations in genes encoding PBPs and β-lactam resistance in *L. salivarius* strains. Among the identified HMW PBPs—FtsI, PBP2x-like, and PBP1A—numerous aa alterations were detected in FtsI and the PBP2x-like protein, with the data suggesting a potentially greater contribution of FtsI in the development of resistance. The elevated AMP MICs observed in some phenotypically susceptible strains, together with their partial PBP alteration profiles, further support the hypothesis that even moderate increases in β-lactam MICs may be associated with structural changes in PBPs. However, the role of the identified alterations remains to be experimentally confirmed. Further studies are therefore needed to clarify their contribution to the observed resistance phenotype in *L. salivarius*. Despite this limitation, the findings presented here provide new insights into the potential role of PBPs in β-lactam susceptibility in *L. salivarius* and highlight the value of analyzing PBP-encoding genes when investigating antimicrobial susceptibility and assessing the safety of strains intended for probiotic use.

## Figures and Tables

**Table 1:** Minimal inhibitory concentration values (mg/L) of beta-lactam antibiotics obtained for the tested *L. salivarius* strains.

Strain	Ampicillin R > 4 mg/L	Amoxicillin + Clavulanic Acid (2:1)	Cephalothin (1G)	Cefuroxime (2G)	Ceftiofur (3G)	Cefotaxime (3G)	Cefepime (4G)	Imipenem
LMG 9476	≤0.5	≤0.5/0.25	1	1	≤0.125	≤2	≤4	≤2
LMG 9477	≤0.5	≤0.5/0.25	2	1	0.25	≤2	≤4	≤2
T2a	≤0.5	≤0.5/0.25	2	2–4	0.25	≤2	≤4	≤2
T11a	≤0.5	≤0.5/0.25	2–4	1	0.25	≤2	≤4	≤2
T7c	4	2/1	4–8	4–8	0.5	≤2	≤4	≤2
T27	2	2/1	4–8	8–16	2–4	≤2	8	≤2
T17f	64	32/16	16	64	16	≥4	≥16	≤2
T17b	64	≥64/32	16–32	32–64	16	≥4	≥16	≤2
T21a	≥128	≥64/32	16	64	16	≥4	≥16	≤2
T25a	≥128	≥64/32	16–32	≥128	64	≥4	≥16	≤2
T28a	32	32/16	8–16	32	4	≥4	≥16	≤2
T31a	64	32/16	8–16	32–64	16	≥4	≥16	≤2

R—resistant; 1G, 2G, 3G, and 4G indicate the generation of cephalosporins.

**Table 2:** Basic information about the WGS of the studied *L. salivarius* strains along with the results of the detection of resistance genes.

Strain	AMP	GenBank Assembly	WGS Size [Mb]	GC Content [%]	Resistance Genes Detected
LMG 9476	S	GCA_053470885.1	2	32.5	none
LMG 9477	S	GCA_029823545.1	2	32.5	none
T2a	S	GCA_054331695.1	2	33	*lnuC*
T17f	R	GCA_054331755.1	2.1	33	*tetL* (2x), *lnuC, tetM, ermB*
T21a	R	GCA_054331715.1	2.1	33	*tetL* (2x), *tetM, ermB*
T25a	R	GCA_048572735.1	2	33	*tetL, lnuC, ermB*

AMP—ampicillin; S—susceptible; R—resistant; WGS—whole genome sequences.

**Table 3:** Amino acid alterations resulting from mutations in the gene encoding FtsI (length 688 aa) in the tested *L. salivarius* strains.

Strain	AMP MIC	Amino Acid Position														
		44	143	192	405	525	585	620	631	659	660	661	662	663	665	673
LMG 9476	≤0.5	Y	K	S	P	S	D	T	I	K	S	S	V	N	N	A
LMG 9477	≤0.5	Y	K	S	P	S	D	T	I	K	S	S	V	N	N	A
T2a	≤0.5	H	E	A	P	S	N	T	I	K	S	S	I	N	N	A
T11a	≤0.5	ND	ND	ND	P	S	N	T	I	K	S	S	V	N	S	A
T7c	4	ND	ND	ND	P	A	D	N	T	K	S	S	V	N	N	A
T27	2	ND	ND	ND	P	A	N	N	T	K	S	S	I	N	N	A
T17f	64	H	E	S	L	A	D	H	N	-	N	G	I	D	N	V
T17b	64	ND	ND	ND	L	A	D	H	N	-	N	G	I	D	N	V
T21a	≥128	Y	E	A	L	A	D	H	N	-	N	G	I	D	N	V
T25a	≥128	H	E	A	L	A	D	H	N	-	N	G	I	D	N	A
T28a	32	ND	ND	ND	P	A	D	H	N	-	N	G	I	D	N	A
T31a	64	ND	ND	ND	P	A	D	H	N	-	N	G	I	D	N	A

AMP MIC—minimal inhibitory concentration of ampicillin; ND—not determined; key aa alterations are highlighted in gray.

**Table 4:** Amino acid substitutions resulting from mutations in the gene encoding the PBP2x-like protein (length 718 aa) in the tested *L. salivarius* strains.

Strain	AMP MIC	Amino Acid Position													
		351	360	437	510	529	536	545	547	574	577	598	603	613	633
LMG 9476	≤0.5	S	Y	G	L	Q	-	Y	F	N	A	V	P	G	V
LMG 9477	≤0.5	S	Y	G	L	Q	-	Y	F	N	A	V	P	G	V
T2a	≤0.5	S	Y	G	L	Q	T	Y	F	N	A	V	S	E	V
T11a	≤0.5	S	Y	G	L	Q	T	Y	F	N	A	V	S	G	V
T7c	4	S	H	A	I	Q	T	Y	L	N	A	V	S	G	V
T27	2	S	Y	A	L	E	T	H	L	N	A	I	S	G	I
T17f	64	P	H	A	I	Q	T	Y	F	N	D	V	S	G	V
T17b	64	P	H	A	I	Q	T	Y	F	N	D	V	S	G	V
T21a	≥128	P	H	A	I	Q	T	Y	F	N	D	V	S	G	V
T25a	≥128	P	H	A	L	Q	T	Y	F	I	D	V	S	G	I
T28a	32	S	H	A	I	Q	T	Y	L	N	A	V	S	G	V
T31a	64	P	H	A	L	Q	T	Y	F	I	D	V	S	G	I

AMP MIC—minimal inhibitory concentration of ampicillin. The aa substitutions found in resistant strains and in susceptible strains with elevated AMP MICs are highlighted in gray.

**Table 5:** Amino acid substitutions resulting from mutations in the gene coding for PBP1A (length 714 aa).

*L. salivarius* Strain	AMP MIC	Amino Acid Position						
		105	106	130	200	220	367	684
LMG 9476	≤0.5	N	A	T	G	K	V	N
LMG 9477	≤0.5	N	A	T	G	E	V	D
T2a	≤0.5	N	A	A	G	E	V	D
T17f	64	M	L	T	G	E	V	D
T21a	≥128	N	A	T	E	E	V	D
T25a	≥128	N	A	A	G	E	I	D

The aa alteration in the transpeptidase domain is highlighted in gray.

**Table 6:** Sequence of primers used for amplification of genes encoding PBPs.

Target Gene Product	Sequence (5′ → 3′)	Annealing Temp. (°C)	Size of PCR Product (bp)
FtsI	F: TCCTGCTGGGGCAATTAATCA R: TCGCTAGATTGAACATCTTTCCAG	56.5	924
PBP2x-like	F: ACAGTTGCATCTGCAATTAATAGTG R: ACAGTCTGATCTCCCTCGACT	56.5	959

## Data Availability

WGS of the *L. salivarius* strains analyzed in this study have been deposited in the GenBank database under the following assembly Acc. Nos.: GCA_054331695.1 (T2a), GCA_054331755.1 (T17f), GCA_054331715.1 (T21a), and GCA_048572735.1 (T25a). The nucleotide sequences of the genes encoding the PBPs FtsI and the PBP2x-like protein from the remaining strains, for which WGS was not performed, have been deposited under Acc. Nos. PZ015971–PZ015982, QB184453–QB184458, and QB184459–QB184463.

## Supplementary Materials

The following supporting information can be downloaded at https://www.mdpi.com/article/10.3390/ijms27219683/s1. Refs. [40,41] are cited in Supplementary Materials file.

## Abbreviations

The following abbreviations are used in this manuscript:

aa	amino acid
AMP	ampicillin
EFSA	European Food Safety Authority
MIC	minimum inhibitory concentration
MRSA	methicillin-resistant *Staphylococcus aureus*
PBP	penicillin-binding protein
