# Supplementary material for: Association Between Alterations in Penicillin-Binding Proteins and β-Lactam Resistance in Ligilactobacillus salivarius
Source: Int J Mol Sci. 2026 Oct 29;27(21):9683. doi: 10.3390/ijms27219683 (PMC13762554; doi:10.3390/ijms27219683)
Supplement: Supplementary file 1 [file ijms-27-09683-s001.zip › ijms-4565722-supplementary.pdf]

# Supplement

**Table S1.** Sequence of the *L. salivarius* FtsI protein (688 aa) in the studied strains, showing variable aa positions (highlighted in yellow) and conserved motifs characteristic of the transpeptidase domain (SXXK, SXN, and KTG).

| Strain | Start | Sequence | End |
|---|---|---|---|
| LMG 9476 | 386 | GSVVKPAMIIGALKSGVITPDNNVLTDMPIKVKDTPVKGSWFNKSGGANVALTASDALMVSSNSYMMQLAMKEAGFK | 462 |
| LMG 9477 | | GSVVKPAMIIGALKSGVITPDNNVLTDMPIKVKDTPVKGSWFNKSGGANVALTASDALMVSSNSYMMQLAMKEAGFK | |
| T2a | | GSVVKPAMIIGALKSGVITPDNNVLTDMPIKVKDTPVKGSWFNKSGGANVALTASDALMVSSNSYMMQLAMKEAGFK | |
| T11a | | GSVVKPAMIIGALKSGVITPDNNVLTDMPIKVKDTPVKGSWFNKSGGANVALTASDALMVSSNSYMMQLAMKEAGFK | |
| T7c | | GSVVKPAMIIGALKSGVITPDNNVLTDMPIKVKDTPVKGSWFNKSGGANVALTASDALMVSSNSYMMQLAMKEAGFK | |
| T27 | | GSVVKPAMIIGALKSGVITPDNNVLTDMPIKVKDTPVKGSWFNKSGGANVALTASDALMVSSNSYMMQLAMKEAGFK | |
| T17b | | GSVVKPAMIIGALKSGVITLDNNVLTDMPIKVKDTPVKGSWFNKSGGANVALTASDALMVSSNSYMMQLAMKEAGFK | |
| T17f | | GSVVKPAMIIGALKSGVITLDNNVLTDMPIKVKDTPVKGSWFNKSGGANVALTASDALMVSSNSYMMQLAMKEAGFK | |
| T21a | | GSVVKPAMIIGALKSGVITLDNNVLTDMPIKVKDTPVKGSWFNKSGGANVALTASDALMVSSNSYMMQLAMKEAGFK | |
| T25a | | GSVVKPAMIIGALKSGVITLDNNVLTDMPIKVKDTPVKGSWFNKSGGANVALTASDALMVSSNSYMMQLAMKEAGFK | |
| T28a | | GSVVKPAMIIGALKSGVITPDNNVLTDMPIKVKDTPVKGSWFNKSGGANVALTASDALMVSSNSYMMQLAMKEAGFK | |
| T31a | | GSVVKPAMIIGALKSGVITPDNNVLTDMPIKVKDTPVKGSWFNKSGGANVALTASDALMVSSNSYMMQLAMKEAGFK | |
| | | | |
| LMG 9476 | 463 | YSYGATLTMPTSIFKKLRNNFNEFGLGVRTGIDLPGEVSGLEGKSTAKYIGSALDLSYGNYDSYTTIQLAQYASI | 537 |
| LMG 9477 | | YSYGATLTMPTSIFKKLRNNFNEFGLGVRTGIDLPGEVSGLEGKSTAKYIGSALDLSYGNYDSYTTIQLAQYASI | |
| T2a | | YSYGATLTMPTSIFKKLRNNFNEFGLGVRTGIDLPGEVSGLEGKSTAKYIGSALDLSYGNYDSYTTIQLAQYASI | |
| T11a | | YSYGATLTMPTSIFKKLRNNFNEFGLGVRTGIDLPGEVSGLEGKSTAKYIGSALDLSYGNYDSYTTIQLAQYASI | |
| T7c | | YSYGATLTMPTSIFKKLRNNFNEFGLGVRTGIDLPGEVSGLEGKSTAKYIGSALDLSYGNYDAYTTIQLAQYASI | |
| T27 | | YSYGATLTMPTSIFKKLRNNFNEFGLGVRTGIDLPGEVSGLEGKSTAKYIGSALDLSYGNYDAYTTIQLAQYASI | |
| T17b | | YSYGATLTMPTSIFKKLRNNFNEFGLGVRTGIDLPGEVSGLEGKSTAKYIGSALDLSYGNYDAYTTIQLAQYASI | |
| T17f | | YSYGATLTMPTSIFKKLRNNFNEFGLGVRTGIDLPGEVSGLEGKSTAKYIGSALDLSYGNYDAYTTIQLAQYASI | |
| T21a | | YSYGATLTMPTSIFKKLRNNFNEFGLGVRTGIDLPGEVSGLEGKSTAKYIGSALDLSYGNYDAYTTIQLAQYASI | |
| T25a | | YSYGATLTMPTSIFKKLRNNFNEFGLGVRTGIDLPGEVSGLEGKSTAKYIGSALDLSYGNYDAYTTIQLAQYASI | |
| T28a | | YSYGATLTMPTSIFKKLRNNFNEFGLGVRTGIDLPGEVSGLEGKSTAKYIGSALDLSYGNYDAYTTIQLAQYASI | |
| T31a | | YSYGATLTMPTSIFKKLRNNFNEFGLGVRTGIDLPGEVSGLEGKSTAKYIGSALDLSYGNYDAYTTIQLAQYASI | |
| | | | |
| LMG 9476 | 538 | LANKGYKIQPHLLQSIRANGKDGKMGAVKYEVKPNITGVIDVPDSYWDIIHSGMYKVVHGTSQYATGTAMKDINP | 612 |
| LMG 9477 | | LANKGYKIQPHLLQSIRANGKDGKMGAVKYEVKPNITGVIDVPDSYWDIIHSGMYKVVHGTSQYATGTAMKDINP | |
| T2a | | LANKGYKIQPHLLQSIRANGKDGKMGAVKYEVKPNITGVIDVPDSYWNIIHSGMYKVVHGTSQYATGTAMKDINP | |
| T11a | | LANKGYKIQPHLLQSIRANGKDGKMGAVKYEVKPNITGVIDVPDSYWNIIHSGMYKVVHGTSQYATGTAMKDINP | |
| T7c | | LANKGYKIQPHLLQSIRANGKDGKMGAVKYEVKPNITGVIDVPDSYWDIIHSGMYKVVHGTSQYATGTAMKDINP | |
| T27 | | LANKGYKIQPHLLQSIRANGKDGKMGAVKYEVKPNITGVIDVPDSYWNIIHSGMYKVVHGTSQYATGTAMKDINP | |
| T17b | | LANKGYKIQPHLLQSIRANGKDGKMGAVKYEVKPNITGVIDVPDSYWDIIHSGMYKVVHGTSQYATGTAMKDINP | |
| T17f | | LANKGYKIQPHLLQSIRANGKDGKMGAVKYEVKPNITGVIDVPDSYWDIIHSGMYKVVHGTSQYATGTAMKDINP | |
| T21a | | LANKGYKIQPHLLQSIRANGKDGKMGAVKYEVKPNITGVIDVPDSYWDIIHSGMYKVVHGTSQYATGTAMKDINP | |
| T25a | | LANKGYKIQPHLLQSIRANGKDGKMGAVKYEVKPNITGVIDVPDSYWDIIHSGMYKVVHGTSQYATGTAMKDINP | |
| T28a | | LANKGYKIQPHLLQSIRANGKDGKMGAVKYEVKPNITGVIDVPDSYWDIIHSGMYKVVHGTSQYATGTAMKDINP | |
| T31a | | LANKGYKIQPHLLQSIRANGKDGKMGAVKYEVKPNITGVIDVPDSYWDIIHSGMYKVVHGTSQYATGTAMKDINP | |
| | | | |
| LMG 9476 | 613 | AIAAKTGTAETVYKNTDTITLSAISYAPYDDPQVVVVVAFPNLASDKSSVNMNTLKSIYEAYWKDVQSSDGYKTSK | 688 |
| LMG 9477 | | AIAAKTGTAETVYKNTDTITLSAISYAPYDDPQVVVVVAFPNLASDKSSVNMNTLKSIYEAYWKDVQSSDGYKTSK | |
| T2a | | AIAAKTGTAETVYKNTDTITLSAISYAPYDDPQVVVVVAFPNLASDKSSINMNTLKSIYEAYWKDVQSSDGYKTSK | |
| T11a | | AIAAKTGTAETVYKNTDTITLSAISYAPYDDPQVVVVVAFPNLASDKSSVNMSTLKSIYEAYWKD----------- | |
| T7c | | AIAAKTGNAETVYKNTDTTTLSAISYAPYDDPQVVVVVAFPNLASDKSSVNMNTLKSIYEAYWKD----------- | |
| T27 | | AIAAKTGNAETVYKNTDTTTLSAISYAPYDDPQVVVVVAFPNLASDKSSINMNTLKSIYEAYWKD----------- | |
| T17b | | AIAAKTGHAETVYKNTDTNTLSAISYAPYDDPQVVVVVAFPNLASD-NGIDMNTLKSIYEVYWKD----------- | |
| T17f | | AIAAKTGHAETVYKNTDTNTLSAISYAPYDDPQVVVVVAFPNLASD-NGIDMNTLKSIYEVYWKDVQSSDGYKTSK | |
| T21a | | AIAAKTGHAETVYKNTDTNTLSAISYAPYDDPQVVVVVAFPNLASD-NGIDMNTLKSIYEVYWKDVQSSDGYKTSK | |
| T25a | | AIAAKTGHAETVYKNTDTNTLSAISYAPYDDPQVVVVVAFPNLASD-NGIDMNTLKSIYEAYWKDVQSSDGYKTSK | |
| T28a | | AIAAKTGHAETVYKNTDTNTLSAISYAPYDDPQVVVVVAFPNLASD-NGIDMNTLKSIYEAYWKD----------- | |
| T31a | | AIAAKTGHAETVYKNTDTNTLSAISYAPYDDPQVVVVVAFPNLASD-NGIDMNTLKSIYEAYWKD----------- | |

**Table S2.** Sequence of the *L. salivarius* PBP2x-like protein (718 aa) in the tested strains, showing variable aa positions (highlighted in yellow) and conserved motifs characteristic for the transpeptidase domain (SXXK, SXN, KTG)

| | | | |
|---|---|---|---|
| LMG 9476 | 275 | KAASINAVVMDSHTGKIVAASQRPTFDAQTKEGLTNVWRNTLLEDAYEPGSTMKVLTVASAINSGNYDSN | 344 |
| LMG 9477 | | KAASINAVVMDSHTGKIVAASQRPTFDAQTKEGLTNVWRNTLLEDAYEPGSTMKVLTVASAINSGNYDSN | |
| T2a | | KAASINAVVMDSHTGKIVAASQRPTFDAQTKEGLTNVWRNTLLEDAYEPGSTMKVLTVASAINSGNYDSN | |
| T11a | | -------------------------------------------------------------------DSN | |
| T7c | | -------------------------------------------------------------------DSN | |
| T27 | | -------------------------------------------------------------------DSN | |
| T17b | | -------------------------------------------------------------------DSN | |
| T17f | | KAASINAVVMDSHTGKIVAASQRPTFDAQTKEGLTNVWRNTLLEDAYEPGSTMKVLTVASAINSGNYDSN | |
| T21a | | KAASINAVVMDSHTGKIVAASQRPTFDAQTKEGLTNVWRNTLLEDAYEPGSTMKVLTVASAINSGNYDSN | |
| T25a | | KAASINAVVMDSHTGKIVAASQRPTFDAQTKEGLTNVWRNTLLEDAYEPGSTMKVLTVASAINSGNYDSN | |
| T28a | | -------------------------------------------------------------------DSN | |
| T31a | | -------------------------------------------------------------------DSN | |
| LMG 9476 | 345 | ATYRSGSYVIDGSTVYDWNRSGWGNITVKEGFMRSSNAVMAQLEQKMGKKTWMSYIRKFGLLRPVGAGLGAESSGN | 420 |
| LMG 9477 | | ATYRSGSYVIDGSTVYDWNRSGWGNITVKEGFMRSSNAVMAQLEQKMGKKTWMSYIRKFGLLRPVGAGLGAESSGN | |
| T2a | | ATYRSGSYVIDGSTVYDWNRSGWGNITVKEGFMRSSNAVMAQLEQKMGKKTWMSYIRKFGLLRPVGAGLGAESSGN | |
| T11a | | ATYRSGSYVIDGSTVYDWNRSGWGNITVKEGFMRSSNAVMAQLEQKMGKKTWMSYIRKFGLLRPVGAGLGAESSGN | |
| T7c | | ATYRSGSYVIDGSTVHDWNRSGWGNITVKEGFMRSSNAVMAQLEQKMGKKTWMSYIRKFGLLRPVGAGLGAESSGN | |
| T27 | | ATYRSGSYVIDGSTVYDWNRSGWGNITVKEGFMRSSNAVMAQLEQKMGKKTWMSYIRKFGLLRPVGAGLGAESSGN | |
| T17b | | ATYRSGPYVIDGSTVHDWNRSGWGNITVKEGFMRSSNAVMAQLEQKMGKKTWMSYIRKFGLLRPVGAGLGAESSGN | |
| T17f | | ATYRSGPYVIDGSTVHDWNRSGWGNITVKEGFMRSSNAVMAQLEQKMGKK-WMSYIRKFGLLRPVGAGLGAESSGN | |
| T21a | | ATYRSGPYVIDGSTVHDWNRSGWGNITVKEGFMRSSNAVMAQLEQKMGKKTWMSYIRKFGLLRPVGAGLGAESSGN | |
| T25a | | ATYRSGPYVIDGSTVHDWNRSGWGNITVKEGFMRSSNAVMAQLEQKMGKKTWMSYIRKFGLLRPVGAGLGAESSGN | |
| T28a | | ATYRSGSYVIDGSTVHDWNRSGWGNITVKEGFMRSSNAVMAQLEQKMGKKTWMSYIRKFGLLRPVGAGLGAESSGN | |
| T31a | | ATYRSGPYVIDGSTVHDWNRSGWGNITVKEGFMRSSNAVMAQLEQKMGKKTWMSYIRKFGLLRPVGAGLGAESSGN | |
| LMG 9476 | 421 | INYTYAFDQANTAYGQGIDVTVIQMMQAFSAIANKGKMVKPRLVDKIVDPNTGKTVYKSKTQVVGKPITAKTSKKV | 496 |
| LMG 9477 | | INYTYAFDQANTAYGQGIDVTVIQMMQAFSAIANKGKMVKPRLVDKIVDPNTGKTVYKSKTQVVGKPITAKTSKKV | |
| T2a | | INYTYAFDQANTAYGQGIDVTVIQMMQAFSAIANKGKMVKPRLVDKIVDPNTGKTVYKSKTQVVGKPITAKTSKKV | |
| T11a | | INYTYAFDQANTAYGQGIDVTVIQMMQAFSAIANKGKMVKPRLVDKIVDPNTGKTVYKSKTQVVGKPITAKTSKKV | |
| T7c | | INYTYAFDQANTAYGQAIDVTVIQMMQAFSAIANKGKMVKPRLVDKIVDPNTGKTVYKSKTQVVGKPITAKTSKKV | |
| T27 | | INYTYAFDQANTAYGQAIDVTVIQMMQAFSAIANKGKMVKPRLVDKIVDPNTGKTVYKSKTQVVGKPITAKTSKKV | |
| T17b | | INYTYAFDQANTAYGQAIDVTVIQMMQAFSAIANKGKMVKPRLVDKIVDPNTGKTVYKSKTQVVGKPITAKTSKKV | |
| T17f | | INYTYAFDQANTAYGQAIDVTVIQMMQAFSAIANKGKMVKPRLVDKIVDPNTGKTVYKSKTQVVGKPITAKTSKKV | |
| T21a | | INYTYAFDQANTAYGQAIDVTVIQMMQAFSAIANKGKMVKPRLVDKIVDPNTGKTVYKSKTQVVGKPITAKTSKKV | |
| T25a | | INYTYAFDQANTAYGQAIDVTVIQMMQAFSAIANKGKMVKPRLVDKIVDPNTGKTVYKSKTQVVGKPITAKTSKKV | |
| T28a | | INYTYAFDQANTAYGQAIDVTVIQMMQAFSAIANKGKMVKPRLVDKIVDPNTGKTVYKSKTQVVGKPITAKTSKKV | |
| T31a | | INYTYAFDQANTAYGQAIDVTVIQMMQAFSAIANKGKMVKPRLVDKIVDPNTGKTVYKSKTQVVGKPITAKTSKKV | |
| LMG 9476 | 497 | LDMMEAVVYDEKGLGQDYAIDGYKIAAKTGTAQIANSNG-GYLSGSSNYIFSVVGMAPADNPRYIMYLTVKQPKS | 571 |
| LMG 9477 | | LDMMEAVVYDEKGLGQDYAIDGYKIAAKTGTAQIANSNG-GYLSGSSNYIFSVVGMAPADNPRYIMYLTVKQPKS | |
| T2a | | LDMMEAVVYDEKGLGQDYAIDGYKIAAKTGTAQIANSNGTGYLSGSSNYIFSVVGMAPADNPRYIMYLTVKQPKS | |
| T11a | | LDMMEAVVYDEKGLGQDYAIDGYKIAAKTGTAQIANSNGTGYLSGSSNYIFSVVGMAPADNPRYIMYLTVKQPKS | |
| T7c | | LDMMEAVVYDEKGIGQDYAIDGYKIAAKTGTAQIANSNGTGYLSGSSNYILSVVGMAPADNPRYIMYLTVKQPKS | |
| T27 | | LDMMEAVVYDEKGLGQDYAIDGYKIAAKTGTAEIANSNGTGYLSGSSNHILSVVGMAPADNPRYIMYLTVKQPKS | |
| T17b | | LDMMEAVVYDEKGIGQDYAIDGYKIAAKTGTAQIANSNGTGYLSGSSNYIFSVVGMAPADNPRYIMYLTVKQPKS | |
| T17f | | LDMMEAVVYDEKGIGQDYAIDGYKIAAKTGTAQIANSNGTGYLSGSSNYIFSVV-MAPADNPRYIMYLTVKQPKS | |
| T21a | | LDMMEAVVYDEKGIGQDYAIDGYKIAAKTGTAQIANSNGTGYLSGSSNYIFSVVGMAPADNPRYIMYLTVKQPKS | |
| T25a | | LDMMEAVVYDEKGLGQDYAIDGYKIAAKTGTAQIANSNGTGYLSGSSNYIFSVVGMAPADNPRYIMYLTVKQPKS | |
| T28a | | LDMMEAVVYDEKGIGQDYAIDGYKIAAKTGTAQIANSNGTGYLSGSSNYILSVVGMAPADNPRYIMYLTVKQPKS | |
| T31a | | LDMMEAVVYDEKGLGQDYAIDGYKIAAKTGTAQIANSNGTGYLSGSSNYIFSVVGMAPADNPRYIMYLTVKQPKS | |
| LMG 9476 | 572 | FGNNDATKNLSTIFNPIMKKVLDSSQVNNTNPGTVKVENVVGNSVDDAKDKISKQGLVPVVVGSGDKVEKQSVEG | 646 |
| LMG 9477 | | FGNNDATKNLSTIFNPIMKKVLDSSQVNNTNPGTVKVENVVGNSVDDAKDKISKQGLVPVVVGSGDKVEKQSVEG | |
| T2a | | FGNNDATKNLSTIFNPIMKKVLDSSQVNNTNSGTVKVENVVENSVDDAKDKISKQGLVPVVVGSGDKVEKQSVEG | |
| T11a | | FGNNDATKNLSTIFNPIMKKVLDSSQVNNTNSGTVKVENVVGNSVDDAKDKISKQGLVPVVVGSGDKVEKQSVE- | |
| T7c | | FGNNDATKNLSTIFNPIMKKVLDSSQVNNTNSGTVKVENVVGNSVDDAKDKISKQGLVPVVVGSGDKVEKQSVE- | |
| T27 | | FGNNDATKNLSTIFNPIMKKVLDSSQINNTNSGTVKVENVVGNSVDDAKDKISKQGLVPVVIGSGDKVEKQSVE- | |
| T17b | | FGNNDDTKNLSTIFNPIMKKVLDSSQVNNTNSGTVKVENVVGNSVDDAKDKISKQGLVPVVVGSGDKVEKQSVE- | |
| T17f | | -GNNDDTKNLSTIFNPIMKKVLDSSQVNNTNSGTVKVENVVGNSVDDAKDKISKQGLVPVVVGSGDKVEKQSVEG | |
| T21a | | FGNNDDTKNLSTIFNPIMKKVLDSSQVNNTNSGTVKVENVVGNSVDDAKDKISKQGLVPVVVGSGDKVEKQSVEG | |
| T25a | | FGINDDTKNLSTIFNPIMKKVLDSSQVNNTNSGTVKVENVVGNSVDDAKDKISKQGLVPVVIGSGDKVEKQSVEG | |
| T28a | | FGNNDATKNLSTIFNPIMKKVLDSSQVNNTNSGTVKVENVVGNSVDDAKDKISKQGLVPVVVGSGDKVEKQSVE- | |
| T31a | | FGINDDTKNLSTIFNPIMKKVLDSSQVNNTNSGTVKVENVVGNSVDDAKDKISKQGLVPVVIGSGDKVEKQSVE- | |
| LMG 9476 | 647 | DQTVISGEKVLLLTNGTKTMPDINGWSRNDIAKLADLTGITVNYTGSGYAYYQSIAAGGALKKSDIVEVKLK | 718 |
| LMG 9477 | | DQTVISGEKVLLLTNGTKTMPDINGWSRNDIAKLADLTGITVNYTGSGYAYYQSIAAGGALKKSDIVEVKLK | |
| T2a | | DQTVISGEKVLLLTNGTKTMPDINGWSRNDIAKLADLTGITVNYTGSGYAYYQSIAAGGALKKSDIVEVKLK | |
| T11a | | ------------------------------------------------------------------------ | |
| T7c | | ------------------------------------------------------------------------ | |
| T27 | | ------------------------------------------------------------------------ | |
| T17b | | ------------------------------------------------------------------------ | |
| T17f | | DQTVISGEKVLLLTNGTKTMPDINGWSRNDIAKLADLTGITVNYTGSGYAYYQSIAAGGALKKSDIVEVKLK | |
| T21a | | DQTVISGEKVLLLTNGTKTMPDINGWSRNDIAKLADLTGITVNYTGSGYAYYQSIAAGGALKKSDIVEVKLK | |
| T25a | | DQTVISGEKVLLLTNGTKTMPDINGWSRNDIAKLADLTGITVNYTGSGYAYYQSIAAGGALKKSDIVEVKLK | |
| T28a | | ------------------------------------------------------------------------ | |
| T31a | | ------------------------------------------------------------------------ | |

**Table S3.** Sequence of the *L. salivarius* PBP1A protein (714 aa) in the tested strains, showing variable aa positions (highlighted in yellow) and conserved motifs characteristic for of the transglycosylase domain (EDxxFxxHxG, STxxxQ, RKxxE, KxxIxxxYxN, and RxxxVL) (red frames) and the transpeptidase domain (SXXK, SXN, and KTG) (black frames)

| | | | |
|---|---|---|---|
| LMG 9476 | 1 | MKIKDFFEKIKDDFLANSRIFGSWFKKKWTRYQGWRWLTIVFLSIFFLISSYGVFLAKKTNVRGLKSALQTS | 72 |
| LMG 9477 | | MKIKDFFEKIKDDFLANSRIFGSWFKKKWTRYQGWRWLTIVFLSIFFLISSYGVFLAKKTNVRGLKSALQTS | |
| T2a | | MKIKDFFEKIKDDFLANSRIFGSWFKKKWTRYQGWRWLTIVFLSIFFLISSYGVFLAKKTNVRGLKSALQTS | |
| T17f | | MKIKDFFEKIKDDFLANSRIFGSWFKKKWTRYQGWRWLTIVFLSIFFLISSYGVFLAKKTNVRGLKSALQTS | |
| T21a | | MKIKDFFEKIKDDFLANSRIFGSWFKKKWTRYQGWRWLTIVFLSIFFLISSYGVFLAKKTNVRGLKSALQTS | |
| T25a | | MKIKDFFEKIKDDFLANSRIFGSWFKKKWTRYQGWRWLTIVFLSIFFLISSYGVFLAKKTNVRGLKSALQTS | |
| LMG 9476 | 73 | TEIYDYKGQKAGALYSQKGTWVPLDKISDNVQNAVLSTEDRNFYHEYGFSITGIGRATLLYAKNKILHRDYI | 144 |
| LMG 9477 | | TEIYDYKGQKAGALYSQKGTWVPLDKISDNVQNAVLSTEDRNFYHEYGFSITGIGRATLLYAKNKILHRDYI | |
| T2a | | TEIYDYKGQKAGALYSQKGTWVPLDKISDNVQNAVLSTEDRNFYHEYGFSITGIGRAALLYAKNKILHRDYI | |
| T17f | | TEIYDYKGQKAGALYSQKGTWVPLDKISDNVQMLVLSTEDRNFYHEYGFSITGIGRATLLYAKNKILHRDYI | |
| T21a | | TEIYDYKGQKAGALYSQKGTWVPLDKISDNVQNAVLSTEDRNFYHEYGFSITGIGRATLLYAKNKILHRDYI | |
| T25a | | TEIYDYKGQKAGALYSQKGTWVPLDKISDNVQNAVLSTEDRNFYHEYGFSITGIGRAALLYAKNKILHRDYI | |
| LMG 9476 | 145 | SGGGSTLTQQLVKNAFLSQEQTFSRKIKEIFIAMQVENDYSKKDILAMYLNNAYFGNGVWGVEDAAEKYFGV | 216 |
| LMG 9477 | | SGGGSTLTQQLVKNAFLSQEQTFSRKIKEIFIAMQVENDYSKKDILAMYLNNAYFGNGVWGVEDAAEKYFGV | |
| T2a | | SGGGSTLTQQLVKNAFLSQEQTFSRKIKEIFIAMQVENDYSKKDILAMYLNNAYFGNGVWGVEDAAEKYFGV | |
| T17f | | SGGGSTLTQQLVKNAFLSQEQTFSRKIKEIFIAMQVENDYSKKDILAMYLNNAYFGNGVWGVEDAAEKYFGV | |
| T21a | | SGGGSTLTQQLVKNAFLSQEQTFSRKIKEIFIAMQVENDYSKKDILAMYLNNAYFENGVWGVEDAAEKYFGV | |
| T25a | | SGGGSTLTQQLVKNAFLSQEQTFSRKIKEIFIAMQVENDYSKKDILAMYLNNAYFGNGVWGVEDAAEKYFGV | |
| LMG 9476 | 215 | HASKLSVPQAATLAGMLKSPTAYNPIDHPQASRDRRNVVLGLMEDNKKLSAEQVSQYQATPMTTNDNYQYTS | 288 |
| LMG 9477 | | HASELSVPQAATLAGMLKSPTAYNPIDHPQASRDRRNVVLGLMEDNKKLSAEQVSQYQATPMTTNDNYQYTS | |
| T2a | | HASELSVPQAATLAGMLKSPTAYNPIDHPQASRDRRNVVLGLMEDNKKLSAEQVSQYQATPMTTNDNYQYTS | |
| T17f | | HASELSVPQAATLAGMLKSPTAYNPIDHPQASRDRRNVVLGLMEDNKKLSAEQVSQYQATPMTTNDNYQYTS | |
| T21a | | HASELSVPQAATLAGMLKSPTAYNPIDHPQASRDRRNVVLGLMEDNKKLSAEQVSQYQATPMTTNDNYQYTS | |
| T25a | | HASELSVPQAATLAGMLKSPTAYNPIDHPQASRDRRNVVLGLMEDNKKLSAEQVSQYQATPMTTNDNYQYTS | |
| LMG 9476 | 289 | SYRYPYYFDAVINEAMNRYGLSETDIMNRGYKIYTSLNQNYQNEMQDDFADSSLFPYNAEDGTKAQGASIAI | 360 |
| LMG 9477 | | SYRYPYYFDAVINEAMNRYGLSETDIMNRGYKIYTSLNQNYQNEMQDDFADSSLFPYNAEDGTKAQGASIAI | |
| T2a | | SYRYPYYFDAVINEAMNRYGLSETDIMNRGYKIYTSLNQNYQNEMQDDFADSSLFPYNAEDGTKAQGASIAI | |
| T17f | | SYRYPYYFDAVINEAMNRYGLSETDIMNRGYKIYTSLNQNYQNEMQDDFADSSLFPYNAEDGTKAQGASIAI | |
| T21a | | SYRYPYYFDAVINEAMNRYGLSETDIMNRGYKIYTSLNQNYQNEMQDDFADSSLFPYNAEDGTKAQGASIAI | |
| T25a | | SYRYPYYFDAVINEAMNRYGLSETDIMNRGYKIYTSLNQNYQNEMQDDFADSSLFPYNAEDGTKAQGASIAI | |
| LMG 9476 | 361 | NPQNGGVAAVVGGREGTHVFRGYNRGTQLVRSPGSTIKPLAVYTPALQSGYHYDTDVKDELKAYGKDKYEPH | 432 |
| LMG 9477 | | NPQNGGVAAVVGGREGTHVFRGYNRGTQLVRSPGSTIKPLAVYTPALQSGYHYDTDVKDELKAYGKDKYEPH | |
| T2a | | NPQNGGVAAVVGGREGTHVFRGYNRGTQLVRSPGSTIKPLAVYTPALQSGYHYDTDVKDELKAYGKDKYEPH | |
| T17f | | NPQNGGVAAVVGGREGTHVFRGYNRGTQLVRSPGSTIKPLAVYTPALQSGYHYDTDVKDELKAYGKDKYEPH | |
| T21a | | NPQNGGVAAVVGGREGTHVFRGYNRGTQLVRSPGSTIKPLAVYTPALQSGYHYDTDVKDELKAYGKDKYEPH | |
| T25a | | NPQNGGIAAVVGGREGTHVFRGYNRGTQLVRSPGSTIKPLAVYTPALQSGYHYDTDVKDELKAYGKDKYEPH | |
| LMG 9476 | 433 | NWNDVYSGEIPMYQALAESKNTSAVWLLNKIGVSKGYKSVEKFGIKLTSDDENLSLALGGLKKGVSPYQMAN | 504 |
| LMG 9477 | | NWNDVYSGEIPMYQALAESKNTSAVWLLNKIGVSKGYKSVEKFGIKLTSDDENLSLALGGLKKGVSPYQMAN | |
| T2a | | NWNDVYSGEIPMYQALAESKNTSAVWLLNKIGVSKGYKSVEKFGIKLTSDDENLSLALGGLKKGVSPYQMAN | |
| T17f | | NWNDVYSGEIPMYQALAESKNTSAVWLLNKIGVSKGYKSVEKFGIKLTSDDENLSLALGGLKKGVSPYQMAN | |
| T21a | | NWNDVYSGEIPMYQALAESKNTSAVWLLNKIGVSKGYKSVEKFGIKLTSDDENLSLALGGLKKGVSPYQMAN | |
| T25a | | NWNDVYSGEIPMYQALAESKNTSAVWLLNKIGVSKGYKSVEKFGIKLTSDDENLSLALGGLKKGVSPYQMAN | |
| LMG 9476 | 505 | AYTAFANGGKLYTAHFITKIVDAQGKVIVDNTENDSKKIMTKKIANEMTSMMMDVYRTGGTGQSASPSGYTI | 576 |
| LMG 9477 | | AYTAFANGGKLYTAHFITKIVDAQGKVIVDNTENDSKKIMTKKIANEMTSMMMDVYRTGGTGQSASPSGYTI | |
| T2a | | AYTAFANGGKLYTAHFITKIVDAQGKVIVDNTENDSKKIMTKKIANEMTSMMMDVYRTGGTGQSASPSGYTI | |
| T17f | | AYTAFANGGKLYTAHFITKIVDAQGKVIVDNTENDSKKIMTKKIANEMTSMMMDVYRTGGTGQSASPSGYTI | |
| T21a | | AYTAFANGGKLYTAHFITKIVDAQGKVIVDNTENDSKKIMTKKIANEMTSMMMDVYRTGGTGQSASPSGYTI | |
| T25a | | AYTAFANGGKLYTAHFITKIVDAQGKVIVDNTENDSKKIMTKKIANEMTSMMMDVYRTGGTGQSASPSGYTI | |
| LMG 9476 | 577 | AGKTGSTESLTASNSSNENDHWYIGYTPDVVVATWVGFDSTKYSLSEEGIRGGASLFKNEMEGILPHTAGNT | 648 |
| LMG 9477 | | AGKTGSTESLTASNSSNENDHWYIGYTPDVVVATWVGFDSTKYSLSEEGIRGGASLFKNEMEGILPHTAGNT | |
| T2a | | AGKTGSTESLTASNSSNENDHWYIGYTPDVVVATWVGFDSTKYSLSEEGIRGGASLFKNEMEGILPHTAGNT | |
| T17f | | AGKTGSTESLTASNSSNENDHWYIGYTPDVVVATWVGFDSTKYSLSEEGIRGGASLFKNEMEGILPHTAGNT | |
| T21a | | AGKTGSTESLTASNSSNENDHWYIGYTPDVVVATWVGFDSTKYSLSEEGIRGGASLFKNEMEGILPHTAGNT | |
| T25a | | AGKTGSTESLTASNSSNENDHWYIGYTPDVVVATWVGFDSTKYSLSEEGIRGGASLFKNEMEGILPHTAGNT | |
| LMG 9476 | 649 | FTVKPASTMVAQESGASSSSSNVWDTFRKAGETLDNSFSKSASDWGSKASSLFDQGKQKLQEWFGQ | 714 |
| LMG 9477 | | FTVKPASTMVAQESGASSSSSNVWDTFRKAGETLDDSFSKSASDWGSKASSLFDQGKQKLQEWFGQ | |
| T2a | | FTVKPASTMVAQESGASSSSSNVWDTFRKAGETLDDSFSKSASDWGSKASSLFDQGKQKLQEWFGQ | |
| T17f | | FTVKPASTMVAQESGASSSSSNVWDTFRKAGETLDDSFSKSASDWGSKASSLFDQGKQKLQEWFGQ | |
| T21a | | FTVKPASTMVAQESGASSSSSNVWDTFRKAGETLDDSFSKSASDWGSKASSLFDQGKQKLQEWFGQ | |
| T25a | | FTVKPASTMVAQESGASSSSSNVWDTFRKAGETLDDSFSKSASDWGSKASSLFDQGKQKLQEWFGQ | |

**Table S4.** GenBank Acc. Nos. of the housekeeping gene sequences of *L. salivarius* strains used for MLST analysis

| gene► (number of known alleles) | ***pstB*** (16) | | ***rpsB*** (12) | | ***nrdB*** (13) | | ***rpoA*** (11) | | ***parB*** (17) | |
|---|---|---|---|---|---|---|---|---|---|---|
| strain▼ | Allele No. | GenBank Acc. No. | Allele No. | GenBank Acc. No. | Allele No. | GenBank Acc. No. | Allele No. | GenBank Acc. No. | Allele No. | GenBank Acc. No. |
| **T2a** | allele 17 new | CM135816.1 | allele 13 new | MT163699; CM135816. | allele 5 | MT192148; CM135816. | allele 6 | MK862013; CM135816. | allele 18 new | MT181939; CM135816. |
| **T7c** | allele 17 new | MT192176 | allele 7 | MT163701 | allele 5 | MT192150 | allele 6 | MK862015 | allele 19 new | MT181941 |
| **T17f** | allele 18 new | MT192177; CM135819. | allele 7 | MT163702; CM135819. | allele 8 | MT192151; CM135819. | allele 12 new | MK862016; CM135819. | allele 20 new | MT181942; CM135819. |
| **T21a** | allele 18 new | CM135817.1 | allele 7 | CM135817.1 | allele 8 | CM135817.1 | allele 13 new | CM135817.1 | allele 20 new | CM135817.1 |
| **T25a** | allele 18 new | CP183830.1 | allele 8 | CP183830.1 | allele 8 | CP183830.1 | allele 6 | CP183830.1 | allele 22 new | CP183830.1 |
| **T27** | allele 18 new | MT192182 | allele 8 | MT163707 | allele 8 | MT192156 | allele 6 | MK862021 | allele 23 new | MT181947 |
| **T31a** | allele 18 new | MT192181 | allele 8 | MT163706 | allele 8 | MT192155 | allele 6 | MK862020 | allele 20 new | MT181946 |

**Table S5.** Sequences of primers used to detect genes coding for β-lactamases

| **Target gene** | **Sequence (5′→ 3′)** | **Annealing temperature (°C)** | **Size of amplification product (bp)** | **Reference** |
|---|---|---|---|---|
| *blaZ* | F: ACTTCAACACCTGCTGCTTTC<br>R: TAGGTTCAGATTGGCCCTTAG | 54 | 238 | Garofalo et al. 2007 |
| *blaTEM* | F: GAGTATTCAACATTTTCGT<br>R: ACCAATGCTTAATCAGTGA | 58 | 445 | Fang et al. 2008 |
| *blaCTX* | F: CGCCGCATACACTATTCTCAGAATGA<br>R: ACGCTCACCGGCTCCAGATTTAT | | 593 | |
| *blaOXA* | F: ATGTGCAGyACCAGTAArGTkATGGC<br>R:TGGGTrAArTArGTsACCAGAAyCAGCG | | 813 | |
| *blaSHV* | F: ACACAATACATATCAACTTCGC<br>R: AGTGTGTTTAGAATGGTGATC | | 237 | |

Garofalo, C.; Vignaroli, C.; Zandri, G.; et al. Direct detection of antibiotic resistance genes in specimens of chicken and pork meat. *Int. J. Food Microbiol.* **2007**, *113*, 75–83.

Fang, H.; Ataker, F.; Hedin, G.; et al. Molecular epidemiology of extended-spectrum beta-lactamases among Escherichia coli isolates collected in a Swedish hospital and its associated health care facilities from 2001 to 2006. *J. Clin. Microbiol.* **2008**, *46*, 707–712.
